# Harnessing deep learning for proteome-scale detection of amyloid signaling motifs

**DOI:** 10.1093/bioinformatics/btaf200

**Published:** 2025-07-15

**Authors:** Krzysztof Pysz, Jakub Gałązka, Witold Dyrka

**Affiliations:** Katedra Inżynierii Biomedycznej, Wydział Podstawowych Problemów Techniki, Politechnika Wrocławska, Wybrzeże Wyspiańskiego 27, 50-370 Wrocław, Poland; Katedra Inżynierii Biomedycznej, Wydział Podstawowych Problemów Techniki, Politechnika Wrocławska, Wybrzeże Wyspiańskiego 27, 50-370 Wrocław, Poland; Wydział Informatyki i Telekomunikacji, Politechnika Wrocławska, Wybrzeże Wyspiańskiego 27, 50-370 Wrocław, Poland; Katedra Inżynierii Biomedycznej, Wydział Podstawowych Problemów Techniki, Politechnika Wrocławska, Wybrzeże Wyspiańskiego 27, 50-370 Wrocław, Poland

## Abstract

**Motivation:**

Amyloid signaling sequences adopt the cross-β fold that is capable of self-replication in the templating process. Propagation of the amyloid fold from the receptor to the effector protein is used for signal transduction in the immune response pathways in animals, fungi, and bacteria. So far, a dozen of families of amyloid signaling motifs (ASMs) have been classified. Unfortunately, due to the wide variety of ASMs it is difficult to identify them in large protein databases available, which limits the possibility of conducting experimental studies. To date, various deep learning (DL) models have been applied across a range of protein-related tasks, including domain family classification and the prediction of protein structure and protein–protein interactions.

**Results:**

In this study, we develop tailor-made bidirectional LSTM and BERT-based architectures to model ASM, and compare their performance against a state-of-the-art machine learning grammatical model. Our research is focused on developing a discriminative model of generalized ASMs, capable of detecting ASMs in large datasets. The DL-based models are trained on a diverse set of motif families and a global negative set, and used to identify ASMs from remotely related families. We analyze how both models represent the data and demonstrate that the DL-based approaches effectively detect ASMs, including novel motifs, even at the genome scale.

**Availability and implementation:**

The models are provided as a Python package, asmscan-bilstm, and a Docker image at https://github.com/chrispysz/asmscan-proteinbert-run. The source code can be accessed at https://github.com/jakub-galazka/asmscan-bilstm and https://github.com/chrispysz/asmscan-proteinbert. Data and results are at https://github.com/wdyrka-pwr/ASMscan.

## 1 Introduction

Amyloid signaling motifs (ASMs) are short amino acid sequences (around 25 amino acid long) facilitating aggregation into a polymeric fibrillary β-sheet-rich structure, termed amyloid fold (Eichner and Radford 2011, Riek and Eisenberg 2016). Amyloid folds are capable of propagation by imposing their conformation on other proteins through a process of templating (self-replication) and protein-to-protein transmission *in vivo* (Seuring *et al.* 2012, Saupe 2020). Thus, they behave similarly to self-propagating prions. Depending on the perspective, the action of amyloid folds can be considered from a pathological or functional angle. Since amyloid fibrils were initially studied in the context of human protein deposition diseases, such as Alzheimer’s disease, the term is now mainly associated with pathology (Ross and Poirier 2004, Dobson 2017). Regardless of the pathological effects of amyloid fibrils, they also have a number of physiological functions in animals, fungi, and bacteria, including oligomerization and signal transduction (Saupe 2020).

To date, around a thousand signaling amyloids associated with the Nod-like receptors (or NLRs, innate immune system proteins) were identified in filamentous fungi, multicellular bacteria, and archaea (Daskalov *et al.* 2015, Dyrka *et al.* 2020, Wojciechowski *et al.* 2022). Despite sharing the common β-arch structure upon aggregation, ASMs are highly diverse beyond noticeable homology (Dyrka *et al.* 2020, Daskalov *et al.* 2021). There have been identified at least 10 families of bacterial ASMs (Dyrka *et al.* 2020) and four families of fungal ASMs (Wojciechowski *et al.* 2022). Importantly, ASMs work in heterotypic pairs (or triplets), situated in N-termini of NLR receptors or in C-termini of cooperating effectors, typically encoded by collocated genes (Daskalov *et al.* 2015, 2021).

**Figure 1.**
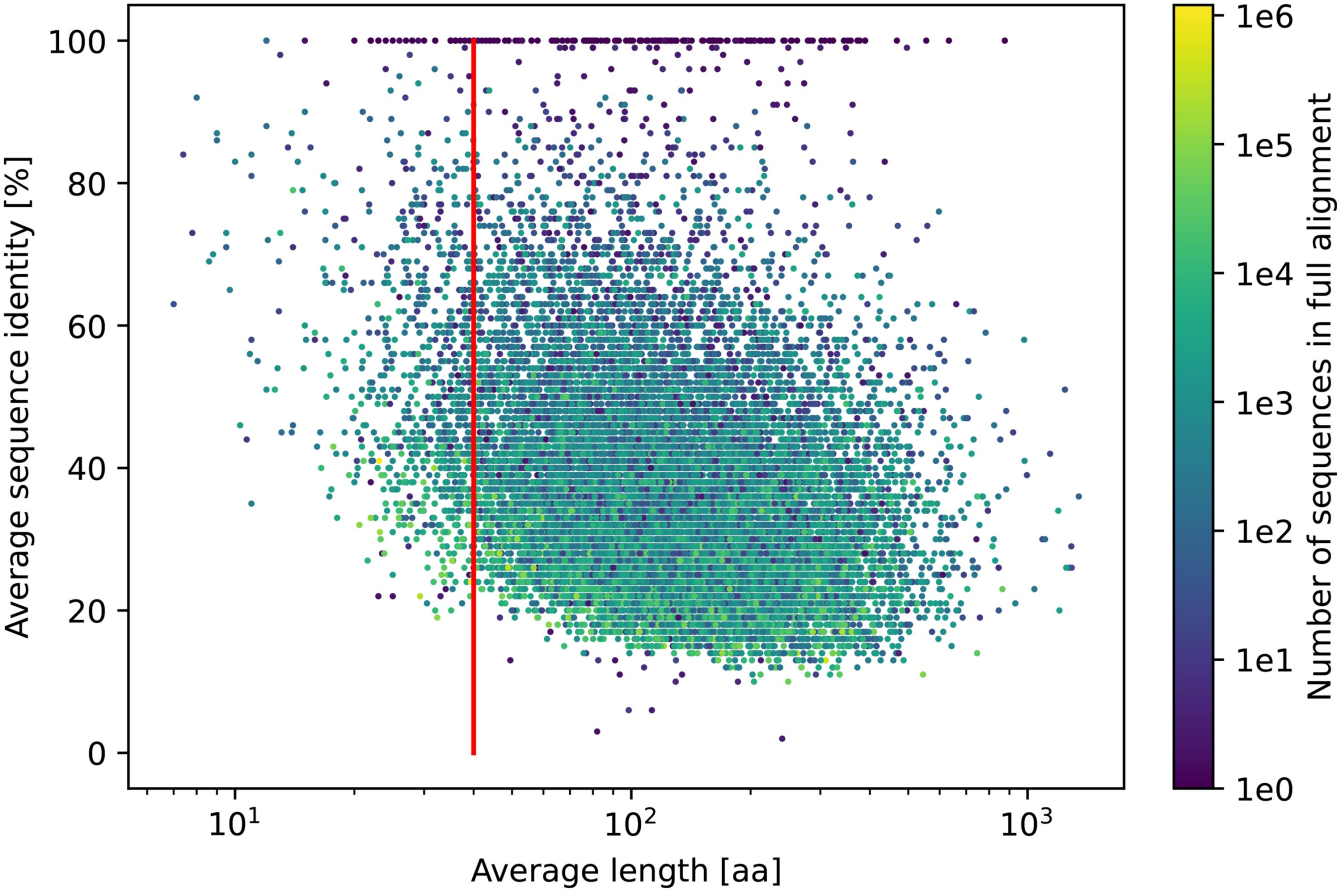
Sequence diversity versus average sequence length in Uniprot-based full alignments for Pfam profiles (Mistry *et al.* 2021). Vertical line corresponds to the average length of 40 amino acids.

For more than two decades, profile HMMs (Eddy 1998, 2011) have remained the standard approach for detection of remote homology between protein domain families and searching for homologous sequences (Potter *et al.* 2018). However, distinctive features of ASMs exacerbate main weaknesses of the method: omission of nonlocal dependencies and limitation to patterns based on homology. Thus, the profile HMM model, which evaluates each alignment position independently (except for indels) is not statistically powerful enough when dealing with short sequences. As a result, in the case of ASMs (and other very short but diverse domains), profile HMMs cannot be sensitive and specific at the same time. For example, in the profile HMM-powered Pfam database (Mistry *et al.* 2021), the median average sequence identity is ca. 50% for 929 families with the average length below 40 in comparison to 33% for 18.5 k longer entries (Fig. 1).

Extensions to the profile HMM framework, such as the Potts model (Weigt *et al.* 2009), can capture pairwise residue–residue correlations in multiple sequence alignment, greatly increasing statistical power. Unfortunately, they have to rely on heuristics to avoid the combinatorial explosion when calculating the sequence-model fit (Lathrop 1994, Muntoni *et al.* 2020, Wilburn and Eddy 2020, Talibart and Coste 2021). Moreover, since inferring the Potts model requires alignment, it is not suitable for modeling functional or structural similarities beyond observable homology.

The aforementioned issues can be addressed to a large extent with more flexible probabilistic context-free grammatical (PCFG) models, which capture some nonlocal dependencies at the expense of cubic computational complexity, which is quite acceptable in the case of short domains (Chomsky 1956, Booth 1969, Booth and Thompson 1973, Dyrka and Nebel 2009, Dyrka *et al.* 2019). Currently, the PCFG-based models are the most effective approach for detecting the ASMs (Dyrka *et al.* 2021, Wojciechowski *et al.* 2022).

Recent advances in the applications of deep learning (DL) techniques in the context of characterizing amino-acid sequences have sparked a remarkable interest in the field of computational biology (Brandes *et al.* 2022). Modern neural networks have been successfully applied to annotation of protein families (Nambiar *et al.* 2020, Brandes *et al.* 2022, Elnaggar *et al.* 2022). For example, ProtENN, which utilizes the ensemble of convolutional neural networks, has enhanced the sequence-level coverage of the Pfam annotations (Bileschi *et al.* 2022). The most dramatic improvement was achieved for the shorter domains (Dohan *et al.* 2021). More recently, PSALM, which incorporates family-specific bidirectional recurrent neural networks on top of the transformer-based general protein model ESM2 (Rives *et al.* 2021), has been reported to improve the domain-level accuracy over HMMER (Sarkar *et al.* 2024). Interestingly, it has been suggested that the ESM2 model represents proteins by storing (local) motifs of pairwise contacts.

In this piece of research, we address a more specific problem by proposing and evaluating two DL-based approaches for identifying ASMs in large protein sequence databases. To this end, we train models of generalized ASMs and compare their performance to the PCFG-based approach (Dyrka *et al.* 2019, 2021, Wojciechowski *et al.* 2022).

## 2 Materials and methods

Following our previous research (Dyrka *et al.* 2019, 2021, Wojciechowski *et al.* 2022), we train models with diverse bacterial ASMs from C-termini of effector proteins, and then test them using bacterial ASMs from N-termini of NLRs and fungal ASMs—as positive samples. As the ASM motifs were extracted such that only the part of motif clearly matching its family pattern was included, this sometimes resulted in truncated motifs (Dyrka *et al.* 2020, Wojciechowski *et al.* 2022). Thus, for testing, we additionally used motifs with two-sided envelopes of 5 and 10 amino acids, and also full-length N- or C-termini of proteins (typically around 100 acid long). In addition, we use two negatives samples: one intended to model entire protein space, derived from (Nambiar *et al.* 2020), and second representing non-ASM effector domains “integrated” into NLR N-termini (Dyrka *et al.* 2021).

### 2.1 Datasets

The BASS datasets consist of 10 families of Bacterial Amyloid Signaling Sequences (BASS) identified in association to NLR proteins in bacteria (Dyrka *et al.* 2020). Training and validation set BASS_C consist of 994 sequences of effector-side motifs with various lengths (21–40 amino acids), nonredundant at nr70. Motif test set BASS_N consists of 181 unique sequences of receptor-side motifs. The motif sequences were delimited according to Supplementary Table 2 in Dyrka *et al.* (2020). In addition, the domain test set BASS_Ndom contains 143 sequences representing the N-termini of NLRs *with* a BASS 1–10 motif (Dyrka *et al.* 2020). These domain sequences were made nonredundant at identity of around 70% (nr70) using CD-HIT (Fu *et al.* 2012). Their lengths vary from 23 to 134 amino acids, with a median length of 70. The sets were previously used in (Dyrka *et al.* 2021) except for BASS_N.

The FASS datasets consist of five families of Fungal Amyloid Signaling Sequences (FASS) identified in association to NLR proteins in fungi. In addition to three motifs associated with HET and HeLo-like effectors (HRAM, σ and PP) (Daskalov *et al.* 2012), the set includes two novel motifs associated with PNP_UDP effectors (PUASM 1–2) (Wojciechowski *et al.* 2022). Notably, the PP motif is homologous to metazoan RHIM and bacterial BASS 3 (Kajava *et al.* 2014, Dyrka *et al.* 2020). Two motif test sets, FASS_C and FASS_N include 92 effector- and 78 receptor-side sequences, respectively, with various lengths (11–30 amino acids), nonredundant at nr90. The motif sequences were delimited according to Supplementary Files 9 and 10 in Wojciechowski *et al.* (2022). In addition, there are two domain test sets, FASS_Cdom and FASS_Ndom, containing 65 effector-side and 67 receptor-side sequences with a FASS motif, respectively. These domain sets consist of 100-amino-acid-long N-termini of NLRs or C-termini of associated effectors, and are nonredundant at nr70.

PB40 is a general negative set based on around 0.5M sequences in UniProtKB/Swiss-Prot adapted from the PRoBERTa project (Nambiar *et al.* 2020). The sequences were cut into overlapping 40-residue long fragments, decimated by a factor of 20, and made nonredundant in a two-stage procedure. They were first roughly clustered with MMseqs2 (Steinegger and Söding 2017) at an identity of 50%, and then with CD-HIT again at nr50 leading to a set of 874 468 sequences. The resulting set was randomly and evenly split into the training and validation set and the test set. The PB40 set is used to approximate the entire space of protein sequences. As such, it may include amyloid signaling sequences.

NLReff is a domain-specific negative set based on a large sample of N-termini of fungal NLR proteins with an integrated nonprion-forming effector domain (Daskalov *et al.* 2020). This test set, previously used in (Dyrka *et al.* 2021), consists of 2411 fragments (nr70) matching the Pfam profiles of the domains (lengths from 41 to 366 amino acids). The NLReff set is used to approximate the background for searching positive test motifs in their typical setting in the N-termini of NLRs.

CsgA is a set of bacterial curli fibers-forming domains involved in biofilm formation and intercellular signaling (Evans and Chapman 2014, Muñoz-Dorado *et al.* 2016). The CsgA domains consist of five imperfect repeats that are typically prone to amyloidogenic aggregation (Szulc *et al.* 2021, 2024). Unrelated to NLRs and structurally distinct from the HET-s fibrils (Wasmer *et al.* 2008, van Melckebeke *et al.* 2010), these domains were included as a control to assess the generalization level of our modeling. Proteins were retrieved from UniProt (UniProt Consortium 2021) searching for protein or gene names containing the “csga” term, and clustered with CD-HIT at nr40 resulting in a set of 38 sequences (CsgA, median length 240.5).

### 2.2 Models

Probabilistic grammar is a generative model of sequential categorical data (Booth 1969, Booth and Thompson 1973). Context-free grammars are a subclass suitable to represent branching and nested dependencies in sequences (ch56). Probabilities of PCFG rules can be estimated solely from the positive training set of sequences, typically with the Inside-Outside (IO) algorithm (Lari and Young 1990, Dyrka *et al.* 2021). In this piece of research, we use an ensemble of six PCFG in the Chomsky Form with Contacts, which is suited to efficiently represent pairwise dependencies between amino acids (Chomsky 1959, Dyrka *et al.* 2019, Pyzik *et al.* 2019). Each model, made with 37 syntactic variables, is parameterized with 85 310 trainable rule probabilities. The PCFG models were inferred using the IO algorithm from the BASS_C set without additional contact constraints (Dyrka *et al.* 2021), available as Supplementary File 7 in Wojciechowski *et al.* (2022). With the PAM 10 smoothing applied to the models (Dyrka *et al.* 2021), the number of rules with probability above 1e−5 is only 1400, on average. This ensemble grammatical BASS model was shown to outperform other approaches in detection of NLR-related amyloid motifs (Dyrka *et al.* 2021) and was successfully applied in searching for novel ASM motifs (Wojciechowski *et al.* 2022).

**Figure 2.**
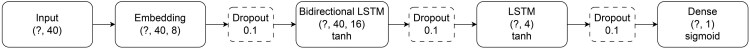
The bidirectional LSTM model architecture consists of two hidden layers. It processes a batch (?) of short protein input sequences of length 40, beginning with an embedding layer that maps inputs to dense representations of size 8. The first hidden layer is a bidirectional LSTM, where each direction has 8 units, resulting in a total of 16 units. It uses tanh activation to capture contextual dependencies from both directions of the sequence. The second hidden layer is a unidirectional LSTM with 4 units and tanh activation, further refining the sequence representation. The output layer is a dense unit with a sigmoid activation, producing the probability of detecting an ASM within the sequence. Dropout layers (10%) are applied between the embedding, bidirectional LSTM, and unidirectional LSTM layers to regulate the model and prevent overfitting.

Long short-term memory (LSTM) is a recurrent neural network architecture designed to address the vanishing gradient problem (Hochreiter and Schmidhuber 1997). By employing memory cells and various gates, the LSTM units are able to retain information over long sequences and selectively update or forget information as needed. LSTM’s ability to capture long-distance relationships makes it well-suited for identifying structural folding patterns and functional motifs that can span considerable distances in protein sequences (Alley *et al.* 2019; Strodthoff *et al*. 2020). For this research, we developed a tailor-made lightweight BiLSTM model, shown in Fig. 2. The architecture incorporates layers with opposite directions of information flow, allowing the model to analyze protein sequences in both forward and backward directions. The outputs from the last units of the forward and backward BiLSTM layers are concatenated, integrating the information from both passes into a single, unified output. The consolidated output from the bidirectional layer is further processed by an additional unidirectional LSTM layer, which refines the sequence representation by simultaneously analyzing both the forward and backward context. Overall, the BiLSTM offers efficient performance for sequential data, being much smaller than more complex architectures and allowing for effective training on moderately sized datasets. Training hyperparameters are listed in Table 1.

**Table 1.** Training hyperparameters of deep learning models.

	BiLSTM	ProteinBERT
Batch size	32	64
Learning rate	1e−3	1e−2; 1e−4
Loss function	Binary cross-entropy	Binary cross-entropy
Number of epochs	30	Early-stopping
		patience = 3
Optimizer	Adam	Adam
Scheduler	None	ReduceLROnPlateau
		factor = 0.25
Trainable parameters	1637	16k; 16M
Weight initialization	Random	Pretrained

See Section 2 for architectural details. ProteinBERT fine-tuning had two phases with different numbers of trainable parameters and learning rates: first, only the classification layer was updated; second, all weights were fine-tuned.

BERT, or Bidirectional Encoder Representations from Transformers, is a Transformer-based model and pretraining approach that enables the capture of comprehensive, context-dependent representations of textual data through a bidirectional self-attention mechanism (Devlin *et al.* 2019). BERT-based models were successfully utilized in tasks such as protein-masked language modeling (Elnaggar *et al.* 2022) and prediction of protein properties (Brandes *et al.* 2022). Usually, these architectures are pretrained on an unlabeled dataset representing the entire protein space in a self-supervised manner (Brandes *et al.* 2022, Elnaggar *et al.* 2022). In this project, we have chosen ProteinBERT (Brandes *et al.* 2022) as the basis of ASM detection model. ProteinBERT extends the regular BERT with specialized architectural elements tailored for protein sequences. It is lightweight for a Transformer-based architecture and well-suited for fine-tuning on downstream tasks, leveraging contextual embeddings pretrained on the large and diverse UniRef90 dataset. In order to adapt ProteinBERT to the ASM detection task, we changed the original model configuration (Brandes *et al.* 2022) by reducing sequence length to match our training data and removing the additional training step with increased sequence length. Fine-tuning was performed in two phases: first, only the classification layer was trained while pretrained layers remained frozen; second, all weights were fine-tuned. In all runs, training completed within 10 epochs. Training hyperparameters are listed in Table 1. The Gene Ontology input of the original ProteinBERT model was skipped by setting all values in the annotation tensor to zero.

### 2.3 Sequence processing

The positive training sequences were sourced from the BASS_C set. As DL models employed in this study necessitate fixed-size sequential input data, thus, to ensure consistency in input dimensions, sequences shorter than 40 amino acids were prepadded using randomly generated amino acid fragments. These fragments, designed to mimic noise, were generated based on the frequency distribution of amino acids observed in the Swiss-Prot database (Gasteiger *et al.* 2003). The negative training sequences sourced from the PB40 set were already standardized to a length of 40 amino acids. In case of PCFG, no padding was applied and no negative data was used for training (Dyrka *et al.* 2021).

Since the sources of test sets, BASS_N, FASS_C, and FASS_N, favored zealous motif excision, motifs with envelopes of 5 or 10 amino acids were also considered in the test setup. In addition, entire N- and C-terminal domains with motifs, BASS_Ndom, FASS_Cdom, and FASS_Ndom, were also used for tests. Since these domains, as well as domains in the negative set NLReff, spanned the length up to 100 or 150 residues, the models processed them using a window of size 40 and step of 1, and then the window with the maximum score (PCFG) or probability (BiLSTM, ProteinBERT) was selected (max-pooling). In case of sequences shorter than the window size, zero postpadding was applied.

### 2.4 Model evaluation

Models were trained and validated in the 6-fold cross-validation process. In the test setting, predictions of the six models were averaged position-wisely, and then—for entire domains only—the max-pooling was applied over the sequence length. These *combined* models were evaluated in terms of Average Precision (AP), Area Under the Receiver Operating Characteristic curve, and Recall for specific False Positive Rate (FPR) thresholds (linearly interpolated from the ROC curve). To better understand how the models interpret the input sequences, particularly when processing the previously unseen data, we applied the Uniform Manifold Approximation and Projection (UMAP) technique (McInnes *et al.* 2020) to visualize the embedding of the highest-scoring subsequences, based on the penultimate layer of each model.

## 3 Results

### 3.1 Discriminative performance

**Figure 3.**
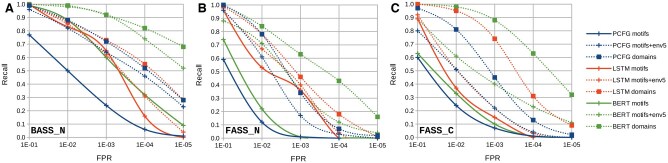
Recall of the combined models versus the FPR on the negative test set sourced from PB40. Positive test samples are (A) BASS_N, (B) FASS_N, and (C) FASS_C. Data points are connected with smooth solid or dotted lines only to improve readability.

The sequences used for training consisted of bacterial effector-side motifs (BASS_C). Initial evaluation was performed using the receptor-side motifs of the same origin (BASS_N and BASS_Ndom). Predictive performance of the three models is presented in Table 2 and Fig. 3A for the four variants of positive sequences extraction. Considering the motifs cut zealously, the BiLSTM and ProteinBERT models clearly outperformed the PCFG model. Including motif envelopes of 5 or 10 amino acids resulted in gradual and significant increases in AP. This established the ranking, with ProteinBERT leading with AP above 0.80, ahead of PCFG (AP up to 0.56) and BiLSTM (0.51). The results for the entire N-terminal domains closely mirrored those for the 10-amino-acid envelope. For ProteinBERT, its maximum performance translated to a Recall approaching 80% at an FPR of $10^{-5}$ (Fig. 3A).

**Table 2.** Average Precision of combined models versus PB40dataset.

Extraction	Model	BASS_N	FASS_N	FASS_C
motifs	PCFG	0.06	0.00	**0.01**
	BiLSTM	0.24	**0.03**	**0.01**
	ProteinBERT	**0.36**	0.00	**0.01**
motifs+env5	PCFG	0.49	0.02	0.03
	BiLSTM	0.36	0.05	0.02
	ProteinBERT	**0.81**	**0.07**	**0.16**
motifs+env10	PCFG	0.56	0.05	0.06
	BiLSTM	0.51	0.06	0.08
	ProteinBERT	**0.85**	**0.24**	**0.30**
domains	PCFG	0.55	0.05	0.09
	BiLSTM	0.59	0.11	0.25
	ProteinBERT	**0.84**	**0.31**	**0.57**

Best results are indicated with bold fonts.

Next, sequences of fungal ASMs were used to assess the generalization ability of the models (Table 2, FASS columns, and Fig. 3B and C). Again, the both neural network-based models outperformed the PCFG method, with the BiLSTM model slightly better for motifs cut zealously, while ProteinBERT showed significant improvement with more generous extraction. However, the models’ performance was notably lower for fungal sequences compared to bacterial sequences, mainly due to low sensitivity at FPRs of $10^{-4}$ and $10^{-5}$ (Fig. 3B and C). The exception was the ProteinBERT model, which achieved a Recall of around 0.4 and 0.6 at a FPR of $10^{-4}$, for the receptor- and effector-side domains, respectively.

### 3.2 Motif position determination

**Figure 4.**
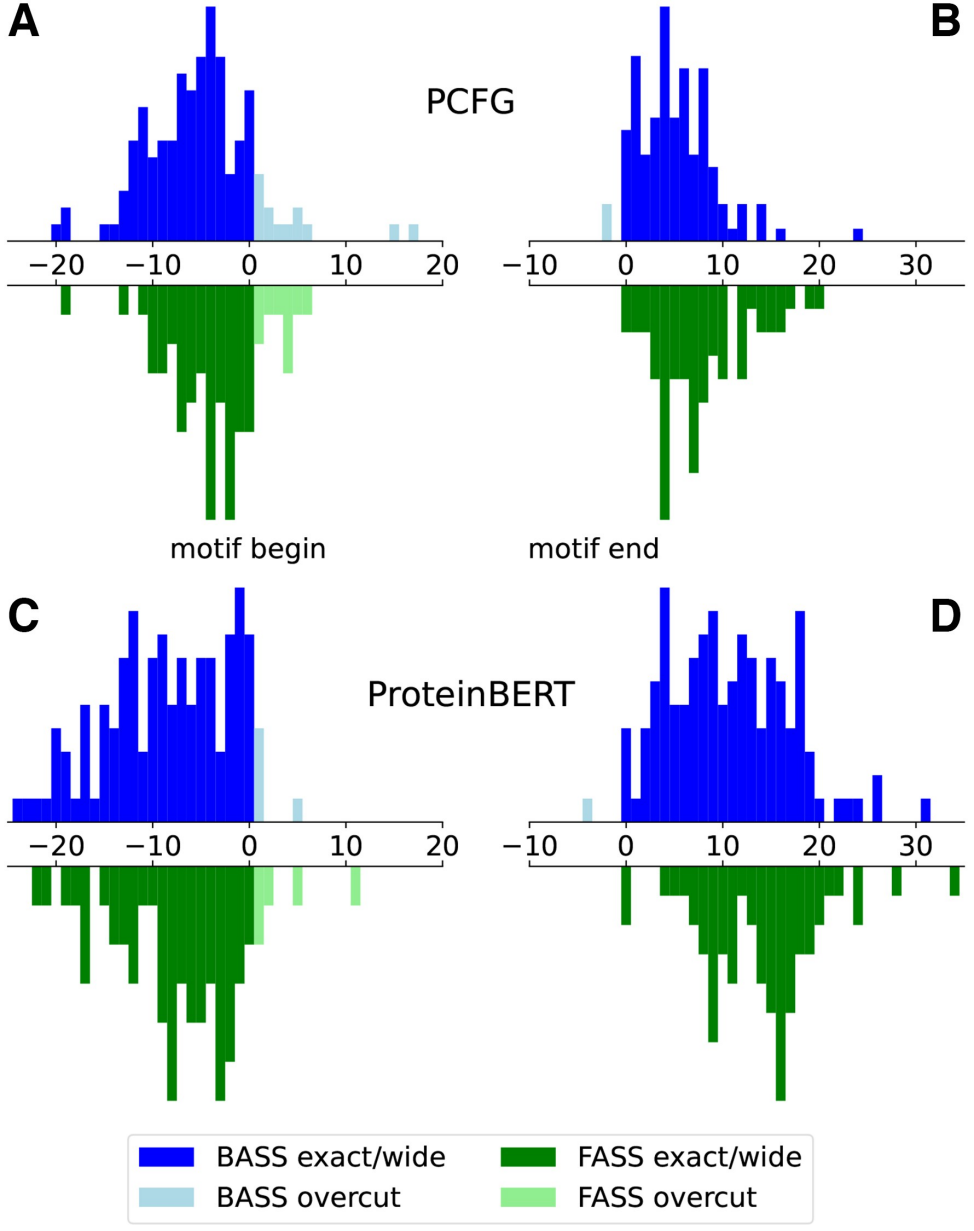
Accuracy of BASS and FASS motif position determination in N-terminal NLR domains (BASS_Ndom, FASS_Ndom) by PCFG (A, B) and ProteinBERT (C, D) combined models. Begin (A, C) and end (B, D) shifts were calculated in relation to positions of rigorously cut test motifs (BASS_N, FASS_N). Histograms include shifts for all best hits regardless of their score/probability except for the only BASS motif which was entirely mispositioned by both methods. Bacterial motifs are shown above the axis, fungal motifs—below the axis.

High discrepancy of PCFG and ProteinBERT models performance on rigorously cut motifs and entire domains prompted us to evaluate the best-scoring window over the domain with regard to the actual motif position. The analysis was conducted on bacterial and fungal receptor-side sequences for PCFG and ProteinBERT models that were the most susceptible to this effect (Fig. 4). For PCFG, we found that on average the best-scoring window covered 97% of the (possibly over) tightly cut motif, typically adding around 5 aa envelope on both sides (Fig. 4A and B). In rare cases when the model truncated the motif, it tended to happen toward the N-terminus. For ProteinBERT, the coverage was even better (Fig. 4C and D). It has to be noted that the ProteinBERT model constantly used the 40-amino-acid-long window, while the PCFG model attempted to find the optimal window ranging from 10 to 40 amino acids. Interestingly, the cuts were generally accurate, even for the optimal windows with low PCFG scores or ProteinBERT probabilities. Only one BASS 1 motif at positions 9–27 in NB-ARC domain-containing protein (GenBank accession PIF86474.1) from *Streptomyces sp.* 76 was entirely outside the best-scoring windows of both methods, despite achieving a high score.

### 3.3 Practical scenarios

We evaluated the models in a real-world scenario with the goal to distinguish amyloid and nonamyloid domains associated with NLR proteins (Dyrka *et al.* 2021, Wojciechowski *et al.* 2022). The positive sets included entire N- and C-termini with bacterial or fungal ASMs, while the negative set included effector domains from the NLR receptor proteins, which did not exhibit known amyloid properties, NLReff (Table 3). ProteinBERT was the best-performing model for the BASS_Ndom and FASS_Ndom sets, achieving 100% and 91% AP and 100% and 78% Recall at an FPR threshold of $10^{-3}$, respectively. For the FASS_Cdom set, the best results were achieved by the PCFG except for the lowest FPR. Even in this most challenging case, AP of the best methods remained above 70% and Recall at an FPR of $10^{-3}$ was close to 50%.

**Table 3.** Evaluation of combined models versus NLReffdataset.

**Pos. sample**	Model	AP	Rc|1e−1	Rc|1e−2	Rc|1e−3
**BASS_Ndom**	PCFG	0.92	0.95	0.85	0.75
**(143 seq.)**	BiLSTM	0.85	0.88	0.77	0.63
	ProteinBERT	**0.96**	**0.99**	**0.93**	**0.87**
**FASS_Ndom**	PCFG	**0.77**	**0.96**	**0.70**	0.42
**(67 seq.)**	BiLSTM	0.58	0.79	0.54	0.27
	ProteinBERT	0.72	0.84	0.66	**0.54**
**FASS_Cdom**	PCFG	0.80	0.91	0.76	0.53
**(65 seq.)**	BiLSTM	0.80	0.97	0.77	0.43
	ProteinBERT	**0.92**	**0.98**	**0.89**	**0.71**

Positive samples consisted of entire N- or C-terminal domains. Negative sample consisted of nonamyloid domains of fungal NLRs. Rc|1e−*k*, interpolated recall at FPR of $10^{-k}$. Best results are indicated with bold fonts.

Finally, we applied the ASM-tuned ProteinBERT for a proteome-wide search. The genome assembly ASM2533192v1 of *Fusarium oxysporum* strain Fo5176 comprises 19 chromosomes and encodes 21 391 proteins. An exhaustive scan using MMSeqs2 (Steinegger and Söding 2017) with Pfam profiles (double HET-s, PF11558, PP (misnamed SesB) PF17046, NACHT_sigma, PF17106, singular HET-S, PF1708) and in-house profiles of HET-s, σ, PP, and PUASM (Wojciechowski *et al.* 2022) identified 23 ASMs, including 17 σ and 6 HET-s motifs. The motifs are encoded by genes clustered in 11 genomic neighborhoods (directly adjacent genes (typically), with maximum separation of 5 kb). This makes it the most ASM-rich fungal assembly annotated at the chromosome level. Accordingly, we used this proteome as a test case to demonstrate one of the intended applications of the ASM-tuned ProteinBERT. Notably, since the model was trained exclusively on bacterial motifs, applying it to a fungal proteome represents a particularly challenging use case.

On a personal laptop, the complete search required 962 min, averaging 2.7 s per sequence or 6 ms per 40-amino-acid fragment. The model returned 124 sequences with hits above its default threshold of 0.5, including 13 σ (distributed across eight genomic neighborhoods) and 1 HET-s motif (Table 4). In addition, a pair of motifs was found in the N-terminus of KAI8417719 (a probable NLR) and the C-terminus of KAI8417720 (associated with SesA domain, Pfam PF17107). This pair exhibited a conserved pattern, NxxxGxQYxxxGxGDQNxVSxxxxQxN, resembling other known ASMs (Wojciechowski *et al.* 2022). Consequently, the motif (provisionally named GDQN) was classified as a *bona fide* ASM. We also tested more stringent detection thresholds, achieving a maximum $F_{1}$ score of 0.33, corresponding to 8 ASMs among the top 21 hits (Table 4). Overall, these results demonstrate that, due to its generalization capability, our ASM detection method can not only largely replace multiple specialized motif searches based on family-specific profiles but also identify motifs belonging to previously unknown ASM families.

**Table 4.** Proteome-wide search using ASM-tuned ProteinBERT in *Fusarium oxysporum* strain Fo5176.

Thr.	#hits	**#** σ	#HET-s	#GDQN	Rec.	Prec.	$F_{1}$
0.500	124	13 (8)	1 (1)	2 (1)	0.64	0.13	0.21
0.750	46	10 (7)	1 (0)	1 (1)	0.48	0.21	0.29
0.845	21	8 (6)	0 (0)	1 (1)	0.36	0.31	0.33

The σ and HET-s counts indicate number of hits annotated as such in Pfam and inhouse profile-based family-specific searches, with numbers in parentheses representing distinct genomic neighborhoods containing these annotated hits (see text for details). The GDQN counts represents a *bona fide* ASM pair identified in this study (see text). Recall and Precision are calculated for the hits above the threshold relative to a total of 25 evidenced ASMs, which include previously annotated σ and HET-s, as well as the two newly discovered GDQN motifs. A threshold of 0.5 is the model’s default, while a threshold of 0.845 was arbitrarily selected to maximize the $F_{1}$ score.

### 3.4 Sequence embedding analysis

**Figure 5.**
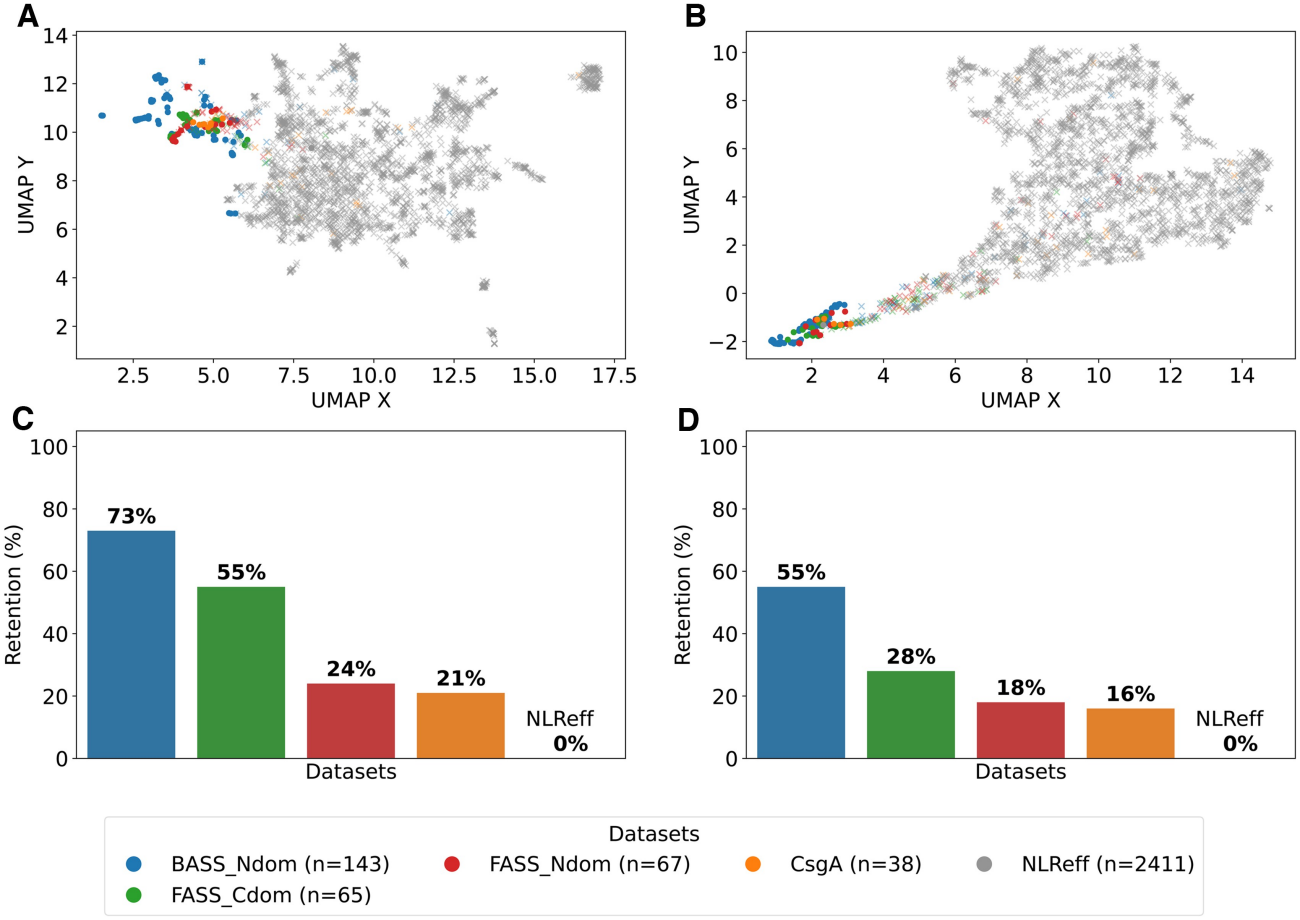
Embeddings of entire N- and C-terminal domains from the BASS_Ndom, FASS_Ndom, and FASS_Cdom sets along with the CsgA and NLReff sets projected to 2-dimensional space using the UMAP technique derived from the final dropout layer of the ProteinBERT (A) and BiLSTM (B) models. Dots represent sequences where ASM was detected, and crosses represent sequences where ASM was not detected, applying a default cut-off of 0.5. The retention plots for the ProteinBERT (C) and BiLSTM (D) models show what percentage of the set are sequences where ASM was detected.

To better understand how the two neural network-based models represent the sequences, we analyzed the embeddings for the highest-scoring 40-amino-acid subsequences, derived from the penultimate layer of each model. The visualizations for the entire domain sets are presented in Fig. 5A and B. In both models, representation of sequences derived from the BASS_Ndom set, which is relatively the most similar to the training set, diverge most from the negative set NLReff. Representations of the FASS_Cdom and FASS_Ndom sequences appear progressively closer to those of the negative background, consistent with decreasing retention (detection) rates (Fig. 5C and D). Additionally, some amyloidogenic CsgA sequences clusters with NLR-related ASMs (with retention rates close to FASS_Ndom), while a substantial portion are more diffusely distributed and intermixed with negative sequences. This suggests that CsgA sequences may display a wider range of characteristics, only some of which align with the positive class. Overall, the projections generated by the ProteinBERT model (Fig. 5A) and the BiLSTM model (Fig. 5B) indicate that both models capture meaningful patterns in the data. More clearer separation of amyloid-positive and amyloid-negative sequences in the ProteinBERT reflects the model’s superior predictive power when entire domains are considered.

**Figure 6.**
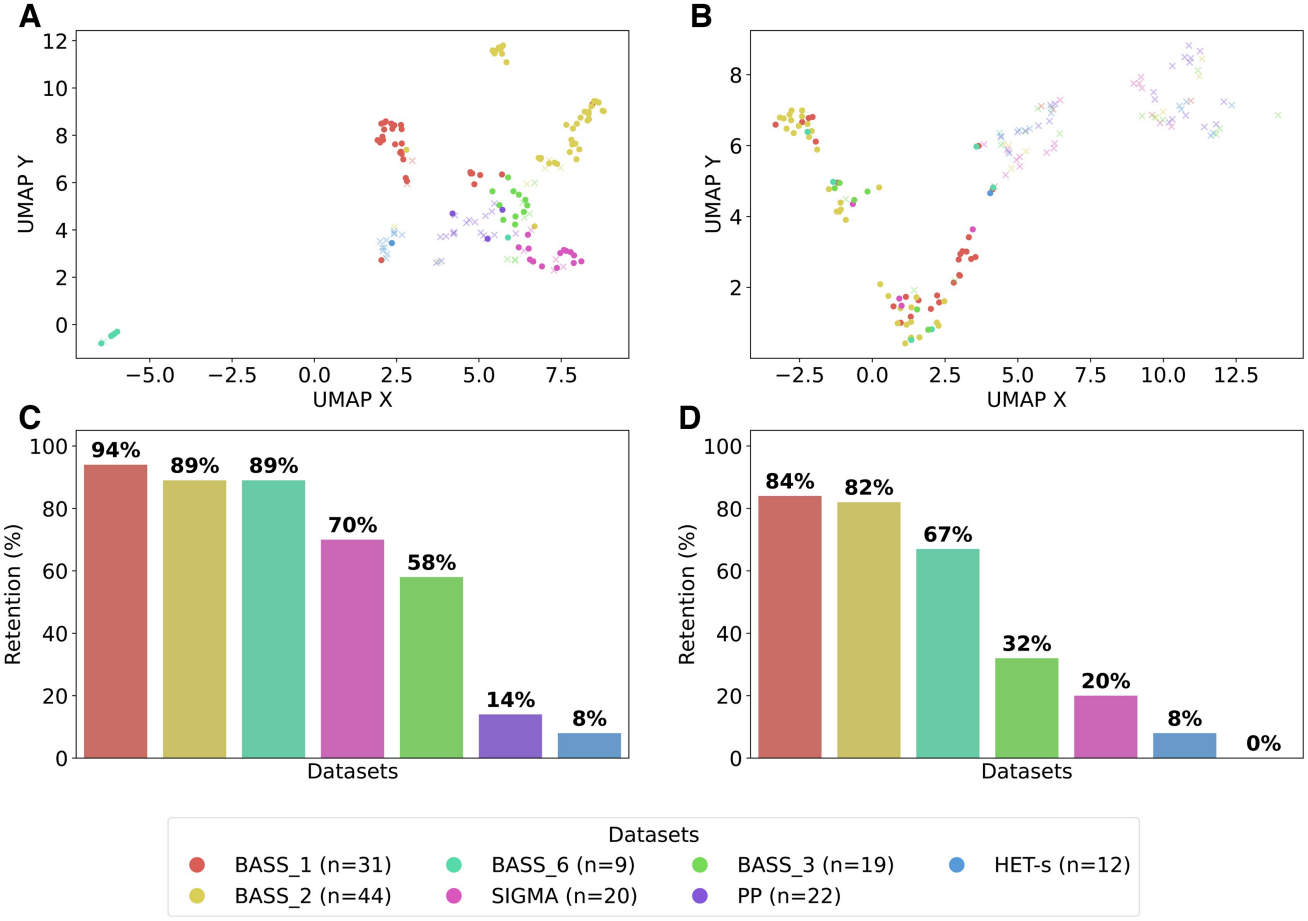
Embedding domains of bacterial and fungal origin with the division into families of ASMs projected to 2-dimensional space using the UMAP technique derived from the final dropout layer of the ProteinBERT (A) and BiLSTM (B) models. Dots represent sequences where ASM was detected, and crosses represent sequences where ASM was not detected, applying a default cut-off of 0.5. The retention plots for the ProteinBERT (C) and BiLSTM (D) models show what percentage of the set are sequences where ASM was detected.

Next, we focused our analysis on the internal representations of the amyloidogenic signaling motifs (Fig. 6). The ProteinBERT embeddings clearly cluster the major families of bacterial and fungal ASMs (Fig. 6A), suggesting that the model effectively detects family-specific signals. Notably, the projection places the two closely related families, BASS 3 and PP, adjacent and at the center of the plot. This positioning aligns with the presumed ancestral origin of this family, as evidenced by its widespread presence across various branches of life including archaea, bacteria (Dyrka *et al.* 2020), fungi (Daskalov *et al.* 2012), and animals (the RHIM motif) (Kajava *et al.* 2014). Interestingly, the clustering of ProteinBERT embeddings is preserved even for false negatives. In contrast, the BiLSTM model produces a projection in which false negatives are distinctly separated from true positives, while sequences from different families are intermixed (Fig. 6B). This observation suggests that the BiLSTM model perceives an essential difference between motifs predicted as positive and negative. The family-specific retention plots (Fig. 6C and D) indicate that both models were most sensitive in recognizing the most abundant families, BASS 1 and BASS 2 families, followed by BASS 4. The σ motif, despite lacking sequence homology to BASS (and therefore to the training set), was the most easily recognized among the fungal motifs, likely due to its longer length and an amino-acid composition resembling that of some BASS (Dyrka *et al.* 2021). Sensitivity for PP and HET-s motifs was poor, while the PP-related BASS 3 was the least detectable among the bacterial motifs. ProteinBERT’s superior performance was most notable for fungal σ (50 pp difference) and bacterial BASS 3 (26 pp). The aforementioned clustering of ProteinBERT embeddings for false-negative hits alongside true-positive hits from the same families suggests that the model could potentially be calibrated to improve sensitivity.

## 4 Discussion and conclusion

We evaluated DL models, including bidirectional LSTM and ProteinBERT-based architectures, for detecting ASMs in proteins. Their performance was compared to a more traditional machine learning approach based on PCFG. The fine-tuned ProteinBERT-based model demonstrated the highest accuracy, particularly in realistic scenarios where motifs are analyzed in the context of entire protein sequences. The model represents a significant advancement in the field, as it has demonstrated practicality not only for identifying ASMs in prefiltered datasets but also in conducting a proteome-wide search. Its performance in ASM annotation is expected to improve further with training on new data encompassing a broader taxonomic scope. Additionally, the model’s characteristics enable optimization of the sensitivity-specificity trade-off through threshold adjustments. The BiLSTM model, on the other hand, performed relatively best in analyzing zealously extracted and truncated sequences, making it more suitable for assessing the amyloid signaling propensity of specific fragments or peptides. Generated without pretraining on large datasets, its performance could likely be significantly enhanced through retraining with more data or by incorporating precomputed contextual embeddings.

Our comparative analysis suggests that ProteinBERT’s superior performance over BiLSTM stems from its context-aware global attention mechanism in the Transformer-based architecture and the residual information retained from pretrained embeddings, captured in a much larger model. Meanwhile, the ranking order between BiLSTM and the PCFG model varied depending on the specific task. This indicates that differences in architectural design and training methodology, including the use of a negative set for training BiLSTM, were insufficient to yield a clear advantage given the similar effective size of the PCFG model.

The ability of models developed in this study to accurately detect and analyze ASMs holds significant promise for both biological research and clinical applications. These tools can help uncover valuable insights into the evolutionary distinctions and similarities among amyloid motif families, ultimately paving the way for developing therapeutic strategies to control or modify amyloid aggregation. The ASM-tuned ProteinBERT enables efficient, proteome-wide searches for candidate ASMs without requiring high sequential similarity to known motifs or task-specific prefiltering based on domain association or genomic colocalization (Wojciechowski *et al.* 2022). For example, it can be used to search for ASMs involved in triggering regulated cell death pathways, providing potential drug targets for combating bacterial and fungal pathogens in humans, animals, and crops (Jones *et al.* 2016, Shlezinger *et al.* 2017). Furthermore, the tool can help identify pairs of microbial and host sequences with potential for disease-related heterotypic cross-interactions (Konstantoulea *et al.* 2022, Elkins *et al.* 2024), thus facilitating downstream analyses. In addition, this study offers a challenging, domain-specific test case for emerging annotation approaches, which aim to surpass traditional profile-based searches.

Conflict of interest: None declared.

## Author contributions

Krzysztof Pysz (Conceptualization [supporting], Data Curation [equal], Formal analysis [equal], Investigation [equal], Methodology [equal], Software [equal], Validation [equal], Vizualization [equal], Writing – Original Draft [equal], Writing – Review & Editing [supporting]), Jakub Gałązka (Conceptualization [supporting], Data Curation [equal], Formal analysis [equal], Investigation [equal], Methodology [equal], Software [equal], Validation [equal], Vizualization [supporting], Writing – Original Draft [equal], Writing – Review & Editing [supporting]), Witold Dyrka (Conceptualization [lead], Data Curation [equal], Formal analysis [equal], Investigation [supporting], Methodology [equal], Supervision [lead], Vizualization [equal], Writing – Original Draft [equal], Writing – Review & Editing [lead])

## Data Availability

The models are provided as a Python package, asmscan-bilstm, and a Docker image at https://github.com/chrispysz/asmscan-proteinbert-run. The source code can be accessed at https://github.com/jakub-galazka/asmscan-bilstm and https://github.com/chrispysz/asmscan-proteinbert. Data and results are at https://github.com/wdyrka-pwr/ASMscan.
